# Dengue fever in Dar es Salaam, Tanzania: clinical features and outcome in populations of black and non-black racial category

**DOI:** 10.1186/s12879-018-3549-z

**Published:** 2018-12-12

**Authors:** Noémie Boillat-Blanco, Belia Klaassen, Zainab Mbarack, Josephine Samaka, Tarsis Mlaganile, John Masimba, Leticia Franco Narvaez, Aline Mamin, Blaise Genton, Laurent Kaiser, Valérie D’Acremont

**Affiliations:** 1 Ifakara Health Institute, Dar es Salaam, United Republic of Tanzania; 2 Swiss Tropical and Public Health Institute, Basel, Switzerland; 3 Department of Sciences, University of Basel, Basel, Switzerland; 4 Infectious Diseases Service, University Hospital of Lausanne (CHUV), Rue du Bugnon 46, 1011 Lausanne, Switzerland; 5 IST clinic, Dar es Salaam, United Republic of Tanzania; 6 Mwananyamala Hospital, Dar es Salaam, United Republic of Tanzania; 7 Arbovirus and imported viral diseases laboratory, National Center of Microbiology, Madrid, Spain; 8 Virology laboratory, University of Geneva, University Hospital of Geneva, Geneva, Switzerland; 9 Department of Ambulatory Care and Community Medicine, University Hospital of Lausanne, Rue du Bugnon 46, 1011 Lausanne, Switzerland

**Keywords:** Dengue, Race, Sub-saharan Africa, Outbreak, Surveillance

## Abstract

**Background:**

Although the incidence of dengue across Africa is high, severe dengue is reported infrequently. We describe the clinical features and the outcome of dengue according to raceduring an outbreak in Dar es Salaam, Tanzania that occurred in both native and expatriate populations.

**Methods:**

Adults with confirmed dengue (NS1 and/or IgM on rapid diagnostic test and/or PCR positive) were included between December 2013 and July 2014 in outpatient clinics. Seven-day outcome was assessed by a visit or a call. Association between black race and clinical presentation, including warning signs, was assessed by logistic regression adjusted for age, malaria coinfection, secondary dengue and duration of symptoms at inclusion. The independent association between demographic and comorbidities characteristics of the patients and severe dengue was evaluated by multivariate logistic regression that included potential confounders.

**Results:**

After exclusion of 3 patients of mixed race, 431 patients with dengue (serotype 2, genotype Cosmopolitan) were included: 241 of black and 190 of non-black race. Black patients were younger (median age 30 versus 41 years; *p* < 0.001) and attended care after a slightly longer duration of symptoms (median of 2.9 versus 2.7 days; *p* = 0.01). Malaria coinfection was not significantly different between black (5%) and non-black (1.6%) patients (*p* = 0.06). The same proportion of patients in both group had secondary dengue (13 and 14%; *p* = 0.78). Among warning signs, only mucosal bleed was associated with race, black race being protective (adjusted OR 0.44; 95% CI 0.21–0.92). Overall, 20 patients (4.7%) presented with severe dengue. Non-black race (adjusted OR 3.9; 95% CI 1.3–12) and previously known diabetes (adjusted OR 43; 95% CI 5.2–361) were independently associated with severe dengue.

**Conclusions:**

Although all patients were infected with the same dengue virus genotype, black race was independently protective against a severe course of dengue, suggesting the presence of protective genetic or environmental host factors among people of African ancestry. The milder clinical presentation of dengue in black patients might partly explain why dengue outbreaks are under-reported in Africa and often mistaken for malaria. These results highlight the need to introduce point-of-care tests, beside the one for malaria, to detect outbreaks and orientate diagnosis.

**Trial registration:**

Clinicaltrials.gov Identifier: NCT01947075, retrospectively registered on the 13 of September 2014.

**Electronic supplementary material:**

The online version of this article (10.1186/s12879-018-3549-z) contains supplementary material, which is available to authorized users.

## Background

Uncontrolled urbanization, human mobility and changes in ecosystems have led to a 30-fold increase of dengue incidence in the last 50 years with geographic expansion to new countries [1]. The America, South-East Asia and western Pacific regions are the most affected [2]. Although dengue is known to circulate in Africa since the nineteenth century, and despite that up to 30 African countries have identified cases, the clinical impact and epidemiology of dengue in this part of the world remains poorly characterized [3]. Current estimates suggest that sub-Saharan Africa carries 16% of the annual worldwide burden, but dengue is often not recognized, and hence under-reported because of its non-specific clinical presentation leading to presumptive diagnosis of malaria. Lack of awareness among clinicians, limited diagnostic capacities and weak surveillance systems may also contribute to underestimation of dengue. A limited number of dengue-infected patients will progress to severe disease which has more specific characteristics and is easier to identify. Although the incidence of dengue across Africa is high, severe dengue has been reported infrequently [3]. To our knowledge, no study has been conducted in Africa evaluating the association between race and dengue presentation and outcome.

Since 2010, dengue outbreaks have been identified in Tanzania [4–6]. We describe the clinical features and outcome of dengue during the first documented outbreak in Dar es Salaam that occurred in 2013–14 in both native and expatriate populations of different races.

## Methods

### Study design and setting

Patients were recruited between December 2013 and July 2014 in four public clinics and one private clinic in Kinondoni, the most populated (1.8 million inhabitants) District of Dar es Salaam, the largest city and economic center of Tanzania.

### Study participants

#### Patients recruited in public and private clinics

A prospective cohort study to document the etiologies of fever was performed between December 2013 and July 2014 at the emergency departments of Mwananyamala Regional Hospital and connected health care facilities (Sinza Hospital, Magomeni Health Centre, Tandale Health Centre). All consecutive adult (age ≥ 18 years) patients presenting with fever (tympanic temperature ≥ 38.0 °C) lasting for 7 days or less at the emergency departments were prospectively screened for inclusion in the study. Simultaneously, consecutive adult patients who presented for medical care in the IST private clinic were screened for dengue in case of clinical suspicion with symptoms lasting for 7 days or less by the medical doctor in charge. Patients diagnosed with dengue (definition below) were included in the present study. The clinical outcome was assessed by a visit or a call 7 days after inclusion in the study.

#### Dengue

Dengue was defined as a positive NS1 antigen or IgM detection with rapid diagnostic test (SD BIOLINE Dengue Duo®) and/or a positive PCR (Fast-track DIAGNOSTICS tropical fever core®).

Secondary dengue infection was defined as evidenced of previous dengue infection as determined by anti-dengue virus IgG detection with rapid diagnostic test during the acute phase (≤5 days of symptoms) of the disease as previously defined [7, 8].

#### Race

Patients were categorized into two groups according to their self-defined race. Those with African ancestry were in the group of patients with black race (black patients from Tanzania and other sub-Saharan African countries) and those without African ancestry were in the group with non-black race (non-black patients from Europe, US, Australia, Asia, Middle East, South Africa).

### Study procedures

#### Data collection

Demographic characteristics, race, comorbidities and symptoms and signs were collected at inclusion using electronic or paper case report forms. Blood pressure was measured with an automated device (Omron® M6). The socio-economic status of black patients was categorized based on indicators of education and wealth. Non-black patients were all expatriates and were considered as having a high socio-economic status.

GPS localization of patients’ home was recorded. Data were entered directly into an open data kit in a personal digital assistant with real-time error, range and consistency checks [9].

#### Laboratory investigations

Rapid diagnostic tests for dengue (SD BIOLINE Dengue Duo®) and malaria (ICT Malaria P.f.®) were systematically performed in patients on site on the day of the interview. Participants enrolled in the public clinics were systematically screened for HIV in accordance with the national algorithm (rapid test, Alere Determine™ HIV-1/2, and for confirmation, a second rapid test, Trinity Biotech Uni-gold™ Recombigen® HIV-1/2). Real-time multiplex PCR (Fast-track DIAGNOSTICS tropical fever core®) targeting dengue virus and Plasmodium was performed in all patients recruited in the public hospitals. Real-time multiplex PCR (Fast-track DIAGNOSTICS Dengue differentiation®) for the detection of dengue virus type 1, 2, 3 and 4 was done in all patients with a positive rapid diagnostic test for dengue in the public and private clinics. RT-PCR analyses were performed at the virology laboratory of the University Hospital of Geneva in Switzerland. Genotyping of dengue virus was performed at the arbovirus and imported viral disease laboratory, National Centre of Microbiology, Madrid, Spain, in a subgroup of randomly selected cases; 4 patients per week (2 in the public and 2 in the private clinic) during the dengue outbreak. A partial and complete envelope gene sequence was obtained using previously described protocols for amplification and sequencing for dengue serotype 2 (DENV-2) [10].

Complete blood count was performed (Horiba Medical ABX Pentra 80 hematology analyzer) at inclusion. Low platelet count was defined as < 100 × 10^9^/L, high hematocrit level as > 45% and low leukocyte count as < 3.5 × 10^9^/L.

#### Dengue warning signs and severe dengue

Dengue warning signs and severe dengue were defined according to WHO recommendations [11]. Warning signs included abdominal pain, persistent vomiting (vomiting during two or more consecutive days), clinical fluid accumulation and mucosal bleed. Three of the seven warning signs, namely liver enlargement, lethargy and increase in hematocrit concurrent with rapid decrease in platelet count were not included as these parameters were not routinely collected. Severe dengue was defined as the presence of at least one of the four following criteria: 1) Circulatory compromise or shock defined as narrow pulse pressure ≤ 20 mmHg or low systolic blood pressure < 90 mmHg, 2) Severe hemorrhage defined as gastrointestinal tract bleeding such as hematemesis, melena or rectorrhagia or menorrhagia, 3) altered mentation defined as a Glasgow coma score of 14 or lower, 4) death within 7 days of follow-up. Severe organ impairment was not a criterion of severe dengue as liver and renal functions were not systematically measured. The clinical outcome and the occurrence of warning signs were recorded at inclusion and by a follow-up visit or call at day seven with the exception of blood count values and blood pressure which were measured at inclusion only.

### Data analysis

Demographic, comorbidities, clinical and laboratory characteristics as well as outcome of dengue patients of black race were compared to those of non-black raceusing Wilcoxon-Mann-Whitney and chi-square tests. In a sub-group analysis, black patients included in the public clinics were compared to those included in the private clinic to account for potential inter-observer variation.

Each warning sign as well as hematocrit and platelets count were evaluated for their association with black race using univariate logistic regression and multivariate logistic regression including potential confounders, i.e. age, malaria coinfection, secondary dengue and duration of symptoms at inclusion.

The independent association between demographic and comorbidities characteristics of the patients and the occurrence of severe dengue was evaluated by multivariate logistic regression that included potential confounders.

Statistical analyses were performed using Stata software (StataCorp, College Station, TX, USA, version 12) and GraphPad Prism 6. Google Earth was used for Fig. 2.

## Results

### Characteristics of patients of black and non-black race

**Fig. 1:**
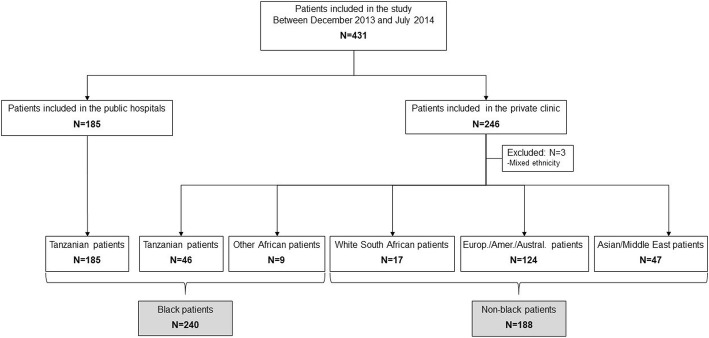
Flow chart of study participants

A total of 431 adult patients with laboratory-confirmed dengue were enrolled in the study. 185 black patients native of Tanzania were enrolled in the public hospitals and 246 patients with different races in the private clinic. Three patients were excluded because of mixed-race leaving 428 patients for analysis (Fig. 1).

**Fig. 2:**
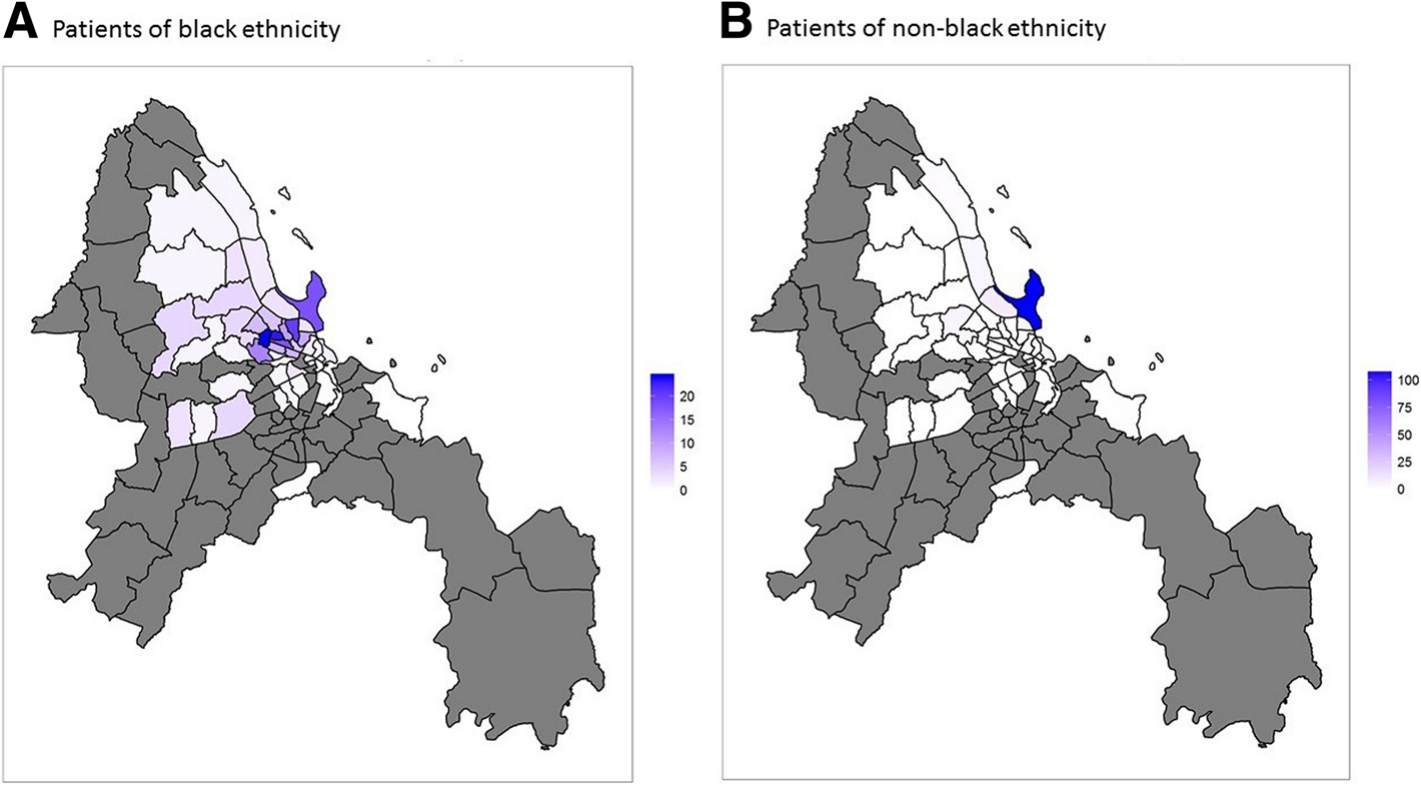
Geolocalisation of dengue patients. **a** Geolocalisation of patients of black race. **b** Geolocalisation of patients of non-black race

Of the 428 dengue patients, 240 were black (185 native Tanzanians recruited in the public clinics; 46 native Tanzanians and 9 from other African countries recruited in the private clinic) and 188 non-black (121 Europeans or Americans, 3 Australians, 39 Asians, 8 from the Middle East and 17 white South Africans recruited in the private clinic). Patients lived in different regions of Dar es Salaam (Fig. 2).

All were infected with same strain of DENV-2 (successful genotyping in 53 out of 87 samples), that belong to Cosmopolitan genotype and was closely related to strains of DENV-2 isolated in Asia in 2013–2014. Strains from the Tanzanian outbreak formed a separate group with Asian strains recently detected in China and Indonesia.

Characteristics of the patients are described in Table 1. Black patients were younger (median age of 30 versus 41 years; *p* < 0.001) and presented to the clinic after a slightly longer duration of symptoms (median of 2.9 versus 2.7 days; *p* = 0.01). Seventy-four percent of black patients and none of the non-black patients had a low socioeconomic status. The prevalence of malaria co-infection was not significantly higher in patients of black (5%) than non-black (1.6%) race (*p* = 0.06). The same proportion of patients in both groups had secondary dengue (13 and 14%, *p* = 0.78). Regarding the clinical presentation of dengue, patients of black race had a higher prevalence of headache (92 and 82%, *p* = 0.003) and myalgia/arthralgia (76% versus 55%, *p* < 0.001) and a lower prevalence of rash (4.2 and 47%, *p* < 0.001). Black patients had a lower prevalence of low blood pressure (0.8% versus 4.8%; p = 0.003), but the same proportion of patients in both groups had narrow pulse pressure (0.8% versus 2.7%; *p* = 0.14). Of note, blood pressure was not measured and assumed to be normal in 129 patients (30%); 36 black patients (15%) and 93 non-black patients (49%).

**Table 1:** Baseline characteristics and day-7 outcome of study participants according to race

	All	Black patients	Non-black patients	*P* value
	*N* = 428	*N* = 240	*N* = 188	
	N(%) or Median (IQR)			
Age, years	35 (27–45)	30 (23–40)	41 (34–49)	< 0.001
Male sex	227 (53)	135 (56)	92 (49)	0.13
Low socioeconomic status	178 (42)	178 (74)	0 (0)	< 0.001
Malaria coinfection	15 (3.5)	12 (5.0)	3 (1.6)	0.06
Pregnancy	8 (1.9)	5 (2.1)	3 (1.6)	0.71
HIV ^a^	11 (2.6)	11 (4.6)	0 (0)	NA
History of diabetes	6 (1.4)	3 (1.3)	3 (1.6)	0.76
Secondary dengue	57 (13.3)	31 (13)	26 (14)	0.78
Symptoms and signs at inclusion				
Duration of symptoms, days; Mean (SD)	2.8 (1.4)	2.9 (1.3)	2.7 (1.6)	0.01
Headache	374 (87)	220 (92)	154 (82)	0.003
Rash	98 (23)	10 (4.2)	88 (47)	< 0.001
Myalgia/Arthralgia	286 (67)	182 (76)	104 (55)	< 0.001
Vomiting	87 (20)	47 (20)	40 (21)	0.67
Systolic blood pressure < 90mmh ^b^	10 (2.3)	1 (0.4)	9 (4.8)	0.003
Pulse pressure ≤ 20 mmHg ^b^	7 (1.6)	2 (0.8)	5 (2.7)	0.14
Altered mental status; Glasgow Coma Score < 15	2 (0.5)	2 (0.8)	0 (0)	0.21
Warning signs at inclusion and within 7 days of follow-up	129 (30)	67 (28)	62 (33)	0.26
Abdominal pain	66 (15.4)	40 (17)	26 (14)	0.42
Persistent vomiting	40 (9.4)	23 (9.6)	17 (9.0)	0.85
Clinical fluid accumulation	4 (0.9)	2 (0.8)	2 (1.1)	0.81
Mucosal bleed	42 (9.7)	14 (5.8)	24 (13)	0.01
Laboratory parameters at inclusion ^c^				
Hemoglobin, mg/l	14 (13–15)	14 (13–15)	14 (12–15)	0.48
Hematocrit, %	42 (39–46)	42 (38–46)	43 (40–46)	0.005
Hematocrit > 45%	120 (28)	54 (23)	66 (35)	0.004
Leukocytes, ×10^9^/L	4.5 (3.1–6.0)	4.6 (3.1–6.2)	4.2 (3.0–5.6)	0.16
Leukocytes < 3.5x10^9^G/L	143 (33)	76 (32)	67 (36)	0.39
Platelets, ×10^9^/L	150 (115–189)	147 (106–195)	11 (122–178)	0.82
Platelets < 100 × 10^9^/L	70 (16)	48 (20)	22 (12)	0.02
Clinical outcome at day 7				
Severe dengue ^d^	20 (4.7)	6 (2.5)	14 (7.5)	0.02
Death	2 (0.5)	2 (0.8)	0 (0)	0.21
Admission	39 (9.1)	21 (8.8)	18 (9.6)	0.77
Intravenous fluid	55 (13)	18 (7.5)	37 (20)	< 0.001

^a^HIV screening was systematically performed in the public clinics only; ^b^ Missing blood pressure measurement in 129 patients (in 36 black and in 93 non-black patients); blood pressure was assumed to be within the normal range if missing; ^c^ Blood count values missing in 18 patients; ^d^ patients who died are included among patients with severe dengue

*Abbreviations*: *NA* Not applicable

Regarding warning signs, the prevalence of mucosal bleed was lower in black patients (5.8% versus 13%; *p* = 0.01). The prevalence of severe dengue was lower in patients of black race (2.5%) compared to those of non-black (7.5%) race (*p* = 0.02).

Compared to black patients included in the private clinic, black patients included in the public clinics were younger (median age of 27 versus 39 years; *p* < 0.001; Additional file 1: Table S1). Regarding the clinical presentation of dengue, black patients included in the public clinic had a higher prevalence of headache (96% versus77%; p < 0.001) and myalgia/arthralgia (80% versus 62%; *p* = 0.006) and a lower prevalence of rash (0% versus 18%;* p *< 0.001) compared to black patients included in the private clinic. However, the same proportion of patients in both groups had hypotension (0.5% versus 0%; *p* = 0.59) and narrow blood pressure (1.1% versus 0%; *p* = 0.44). The proportion of patients with mucosal bleeding (6.0% and 5.5%; *p* = 0.89) and with severe dengue (3.2% and 0%; *p* = 0.18) was not different between black patients included in the public hospitals and those included in the private clinic.

### Association between each warning sign as well as blood count parameters and black race

Except for mucosal bleed, there was no association between warning signs and race. Black race was protective against mucosal bleed (adjusted odds ratio (OR) 0.44; 95% confidence interval (CI) 0.21–0.92) even after adjustment for potential confounders (age, malaria co-infection, secondary dengue and duration of symptoms at inclusion; Table 2). Regarding blood count parameters, high hematocrit was associated with non-black race (adjusted OR 2.0; 95% CI 1.3–3.2) even after adjustment for hemoglobin value (adjusted OR 2.1; 95% CI 1.3–3.3), while the presence of a low platelets count was associated with black race (adjusted OR 1.8; 95% CI 1.0–3.3).

**Table 2:** Association between each warning sign as well as laboratory parameters and black race

	OR or coefficient (95% CI)	*P* value	OR or coefficient (95% CI)	*P* value
	Unadjusted		Adjusted ^a^	
Warning signs at inclusion and within 7 days of follow-up	0.79 (0.52–1.2)	0.26	0.77 (0.49–1.2)	0.26
Abdominal pain	1.2 (0.7–2.1)	0.42	1.2 (0.67–2.1)	0.56
Persistent vomiting	1.1 (0.55–2.1)	1.0	0.95 (0.46–2.0)	0.90
Clinical fluid accumulation	0.78 (0.11–5.6)	0.81	2.0 (0.19–20)	0.58
Mucosal bleed	0.42 (0.21–0.84)	0.01	0.44 (0.21–0.92)	0.03
Laboratory parameters at inclusion ^c^				
Hematocrit	0.97 (0.95–1.0)	0.06	−1.3 (−2.8–0.33) ^b^	0.12
Hematocrit > 45%	0.54 (0.35–0.82)	0.004	0.49 (0.31–0.78) ^b^	0.003
Platelets	1.0 (1.0–1.0)	0.73	4.1 (−9.8–18)	0.58
Platelets < 100 × 10^9^/L	1.9 (1.1–3.3)	0.02	1.8 (1.0–3.3)	0.05

^a^Adjusted for age, malaria coinfection, secondary dengue and duration of symptoms at inclusion

^b^After adjustment for hemoglobin result: hematocrit > 45%: 0.48 (0.30–0.76) *p* = 0.002; hematocrit value: − 1.2 (− 2.7–0.39) *p* = 0.14

^c^Blood count values missing in 18 patients

### Factors associated with severe dengue

Overall, 20 patients out of 428 (4.7%) presented with severe dengue and among them, two died. The criteria classifying them as severe dengue was low systolic blood pressure in 7, narrow pulse pressure in 5, both low systolic pressure and narrow pulse pressure in 2, severe hemorrhage in 3, cardiac failure in 1. The two patients who died presented with altered mental status and one also presented with severe hemorrhage and low systolic blood..

The socio-demographic characteristics (age, sex and socioeconomic status) of patients who presented with severe dengue were similar to those who had a milder course of disease. Secondary dengue was not a factor associated with severe dengue (adjusted OR 1.5; 95% CI 0.4–5.4) (Table 3). Non-black race (adjusted OR 3.9; 95% CI 1.3–12) and known diabetes (adjusted OR 43; 95% CI 5.2–361) were independently associated with severe dengue even after adjustment for potential confounders.

**Table 3:** Factors associated with severe dengue

	Severe dengue	No severe dengue	Univariate analysis	*P *value	Multivariate analysis	*P* value
	*N* = 20	*N* = 408	Unadjusted OR or coefficient (95% CI)		Adjusted OR or coefficient (95% CI)	
Age, years	36 (27–50)	35 (27–45)	1.0 (0.99–1.05)	0.21	0.98 (0.94–1.0)	0.41
Male sex	10 (50)	217 (53)	0.88 (0.36–2.2)	0.78		
Low socioeconomic status	5 (25)	173 (42)	0.45 (0.16–1.3)	0.13		
Black race	6 (30)	234 (57)	0.32 (0.12–0.85)	0.02	0.26 (0.09–0.77)	0.02
Private clinic	14 (70)	229 (56)	1.8 (0.69–4.8)	0.22		
Malaria coinfection	0 (0)	15 (3.7)				
Pregnancy	0 (0)	8 (2.0)				
History of diabetes	3 (15)	3 (0.74)	24 (4.5–127)	< 0.001	43 (5.2–361)	< 0.001
Secondary dengue	3 (15)	554 (13.2)	1.2 (0.33–4.1)	0.82	1.5 (0.40–5.4)	0.56
Warning signs	9 (45)	120 (29)	2.0 (0.79–4.9)	0.14		
Abdominal pain	4 (20)	62 (15)	1.4 (0.45–4.3)	0.56		
Persistent vomiting	4 (20)	36 (8.8)	2.6 (0.82–8.1)	0.1		
Clinical fluid accumulation	1 (5)	3 (0.74)	7.1 (0.71–71.5)	0.1		
Mucosal bleed	5 (25)	33 (8)	3.8)1.3–11)	0.02		
Laboratory parameters ^a^						
Hematocrit, %	43 (41–46)	42 (38–46)	1.0 (0.95–1.1)	0.69		
Hematocrit > 45%	7 (35)	113 (28)	1.4 (0.55–3.61)	0.48		
Platelets, ×10^9^/L	146 (103–186)	150 (116–189)	1.0 (0.99–1.0)	0.71		
Platelets < 100 × 10^9^/L	4 (20)	66 (16)	1.3 (0.42–4.0)	0.65		
Leukocytes, ×10^9^/L	9 (45)	134 (33)	0.99 (0.87–1.1)	0.83		
Leukocytes < 3.5 × 10^9^/L	9 (45)	134 (33)	1.7 (0.68–4.13)	0.27		

^a^Blood count values missing in 18 patients

## Discussion

Although all patients were infected with the same dengue virus genotype, black race was independently protective against a severe course of dengue. These results support the hypothesis of protective genetic or environmental (such as protection after previous exposure to the same dengue strain and cross-protection after exposure to other arboviruses or vaccine) host factors among people of African ancestry.

Our results are in line with previous epidemiologic data showing the absence of severe dengue in Haiti between 1994 and 1996 despite high transmission and by data from the 1981 and 1997 dengue epidemics in Cuba showing a lower rate of hemorrhagic manifestations and hospitalization among subpopulations of African ancestry [12–16]. The Cuban studies also showed that non-black individuals were disproportionately susceptible to dengue [17]. De La Sierra et al. reported a stronger and cross-reactive dengue virus-specific memory CD4^+^ T cell proliferation and interferon-gamma release in white people compared to black people in 80 Cuban donors previously infected with dengue [18]. Blanton et al. also reported an association between African ancestry and a reduced risk of severe dengue in Brazil [3, 19]. In addition, a genetic study from Brazil identified a strong association between a polymorphism in JAK1 and severe dengue and showed a different distribution of mutations by race consistent with the epidemiologic data [20]. In Colombia, an epidemiological study also showed that the Afro-Colombians population had a significantly lower risk of getting dengue and its complications, compared with the non-Afro-Colombians population [21].

In our study, several factors may explain a lower susceptibility to severe disease among the population of black race. Differences in patients’ characteristics, health facility attended and environmental exposure have been identified between black and non-black patients and might have led to some bias in the link between dengue severity and race.

Different patients’ characteristics such as age and socio-economic status were related to race. Black patients were younger and most of them had a low socio-economic status. Older age has been associated with severe dengue in previous reports [11, 22, 23]. However, our analyses were adjusted for age and age was not a factor associated with a severe course of dengue. Low et al. reported that older age was associated with a lower prevalence of myalgia, arthralgia, headache and mucosal bleeding [24]. Age difference between patients of black and non-black race could explain part of the differences observed in the clinical presentation of dengue. Low socio-economic status has also been linked to an increased risk of severe dengue [25]. Blanton et al. addressed both race and socioeconomic factors in a case control study in Brazil and concluded that both ancestry and income are factors associated with severe dengue [19]. In our study, socio-economic status could be a potential confounder as income and race are closely interrelated in this Tanzanian setting. However, it cannot explain the reduced dengue severity among black patients as they had a lower socio-economic status compared to non-black patients. Hemoglobin and hematocrit values might also be linked to race as they are known to be lower in persons of African race [26]. As we do not have values outside of the disease episode, we cannot establish with certainty a link of causality between higher hematocrit value in non-black patients and dengue severity.

The patients were included by two different study teams which might have led to differences in patients’ evaluation. Patients included in the private clinic were mostly non-black (76%) while patients included in the public clinics were all black. However, a different appreciation of the warning signs and dengue severity criteria is unlikely as both study teams were trained to detect severe signs such as mucosal bleed and used the same case report form. Blood pressure was measured with the same device and hematocrit value is not clinician-dependent. Furthermore, when comparing black patients included in the different setting, there was no difference in the prevalence of mucosal bleeding and disease severity.

Black and non-black patients lived in different areas of the city: most non-black patients living in a privileged part of the city while most black patients living in poor and overcrowded wards. However, they were all infected by the same dengue virus strain. This outbreak was not caused by an endemic virus strain that had been circulating in Africa before, but was probably imported from Asia to Tanzania [4, 5]. Black patients were mostly native of Tanzania while non-black patients were mostly expatriate coming from different parts of the world and thus with different environmental exposures in the past. Therefore, the rate of previous infection by another dengue virus serotype might have been different between black and non-black patients and explain a different dengue severity as secondary dengue infection by another virus serotype is a risk factor for severe disease via antibody-dependent infection enhancement while secondary infection by the same serotype induces protection [2]. However, the proportion of patients with secondary dengue was not different between patients of black and non-black race. Furthermore, we do not expect protection in the native black population as only dengue serotype 3 has been described in the past in Tanzania [6, 27]. Exposure or previous vaccination to other flavivirus might have conferred cross-protection against dengue virus. Indeed, black patients native of Tanzania might have been exposed to yellow fever, West Nile or other unrecognized flaviviruses in the past or been vaccinated against yellow fever while Asian patients might have been exposed to or vaccinated against Japanese encephalitis. All flaviviruses are antigenically related and serological cross-reactions between flaviviruses are frequent suggesting that past exposure to a flavivirus might facilitate a secondary booster enhancement effect or cross-protection upon exposure to a different but related virus. In a sero-epidemiologic study, previous exposure to dengue was associated to a reduced severity of yellow fever among military personnel detached in the Ecuadorian Amazonia [28]. A study performed in mice showed a protective effect against dengue viruses induced by the Japanese encephalitis vaccines [29]. Cross-protection between the Japanese encephalitis virus and dengue virus was also detected in humans vaccinated with the Japanese encephalitis vaccine [29]. There has not been any infection with yellow fever in Tanzania for more than 20 years and yellow fever vaccine is not part of the vaccination program in Tanzania. But, most expatriates are vaccinated against yellow fever as recommended for travelers visiting Tanzania. However, there is no evidence that previous yellow fever vaccination has an impact on dengue severity.

Our study has several strengths. Patients were prospectively included during a dengue outbreak and were well characterized. Virus genotyping data allowed ruling out virus characteristics as a factor leading to a difference in disease severity between racial groups. Our study has some limitations. First, in this African setting, race cannot be disentangled from previous exposure to different dengue virus serotypes or other endemic flaviruses as non-black patients were mostly expatriate. Another limitation is the inclusion of patients by two different study team leading in a potential different appreciation of the clinical signs and symptoms of the patients. Third, blood count analysis was only done at inclusion and we could not analyze the rise of hematocrit in the course of the disease.

## Conclusions

In conclusion, this study addressing the relation between race and dengue severity in an African country showed that patients of black race had a lower incidence of severe dengue. This finding suggests the presence of protective genetic host factors among people of African ancestry or environment factors, such as absence of past exposure to other dengue virus serotypes (although the prevalence of secondary dengue in our study was the same) or on the contrary previous exposure to endemic flaviviruses conferring cross-protection against severe dengue. The milder clinical presentation of dengue described in our study might partly explain why dengue outbreaks are under-reported in Africa and often mistaken for malaria. These findings highlight the need to introduce point-of-care tests, beside the one for malaria, to detect outbreaks in a timely manner and, when an outbreak is ongoing, to orientate the diagnosis in febrile patients.

## Additional file

Additional file 1: **Table S1.** Characteristics of black patients included in the private clinic and in the public hospitals. (DOCX 26 kb)

## Abbreviation

DENV-2: Dengue serotype 2
