# Supplementary material for: Dengue fever in Dar es Salaam, Tanzania: clinical features and outcome in populations of black and non-black racial category
Source: BMC Infect Dis. 2018 Dec 12;18:644. doi: 10.1186/s12879-018-3549-z (PMC6292068; doi:10.1186/s12879-018-3549-z)
Supplement: Supplementary file 1 — Table S1. Characteristics of black patients included in the private clinic and in the public hospitals. (DOCX 26 kb) [file 12879_2018_3549_MOESM1_ESM.docx]

Table S1. Characteristics of black patients included in the private clinic and in the public hospitals

|  | Black patients included in public clinics | Black patients included in the private clinic |  |
| --- | --- | --- | --- |
|  | N=185 | N=55 | P value |
|  | N(%) or Median (IQR) | |  |
|  |  |  |  |
| Age, years | 27 (22-35) | 39 (32-51) | <0.001 |
| Male sex | 108 (58%) | 27 (49%) | 0.22 |
| Low socioeconomic status | 166 (90%) | 12 (22%) | <0.001 |
| Malaria coinfection | 10 (5.4%) | 2 (3.6%) | 0.60 |
| Pregnancy | 4 (2.1%) | 1 (1.8%) | 0.88 |
| HIV ^a^ | 10 (5.4%) | 1 (1.8%) | NA |
| History of diabetes | 1 (0.5%) | 2 (3.6%) | 0.07 |
| Secondary dengue | 20 (10.8%) | 11 (20%) | 0.07 |
|  |  |  |  |
| **Symptoms and signs at inclusion** | |  |  |
| Duration of symptoms, days; Mean (SD) | 2.7 (1.1) | 3.3 (1.8) | 0.13 |
| Headache | 178 (96.2) | 43 (76.8) | <0.001 |
| Rash | 0 (0%) | 10 (18%) | <0.001 |
| Myalgia/Arthralgia | 148 (80%) | 34 (62%) | 0.006 |
| Vomiting | 35 (19%) | 12 (22%) | 0.63 |
| Systolic blood pressure <90mmHg ^b^ | 1 (0.5%) | 0 (0%) | 0.59 |
| Pulse pressure ≤20mmHg ^b^ | 2 (1.1%) | 0 (0%) | 0.44 |
| Altered mental status; Glasgow Coma Score <15 | 2 (1.1%) | 0 (0%) | 0.44 |
|  |  |  |  |
| **Warning signs at inclusion and within 7 days of follow-up** | 48 (26%) | 19 (35%) | 0.21 |
| Abdominal pain | 28 (15%) | 12 (22%) | 0.24 |
| Persistent vomiting | 15 (8.1%) | 8 (14.6%) | 0.15 |
| Clinical fluid accumulation | 2 (1.1%) | 0 (0%) | 0.44 |
| Mucosal bleed | 11 (6.0%) | 3 (5.5%) | 0.89 |
|  |  |  |  |
| **Laboratory parameters at inclusion** ^c^ | |  |  |
| Hemoglobin, mg/L | 14 (12-16) | 13 (11-15) | 0.05 |
| Hematocrit, % | 41 (36-46) | 43 39-46) | 0.19 |
| Hematocrit >45% | 41 (22%) | 13 (24%) | 0.82 |
| Leukocytes, G/L | 4.8 (3.3-6.4) | 4.1 (2.7-5.6) | 0.05 |
| Leukocytes <3.5 G/L | 53 (29%) | 23 (42%) | 0.07 |
| Platelets, G/L | 156 (103-198) | 138 (113-178) | 0.42 |
| Platelets <100G/L | 41 (22%) | 7 (13%) | 0.13 |
|  |  |  |  |
| **Clinical outcome at day 7** |  |  |  |
| Severe dengue ^d^ | 6 (3.2%) | 0 (0%) | 0.18 |
| Death | 2 (1.1%) | 0 (0%) | 0.44 |
| Admission | 11 (6.0%) | 10 (18%) | 0.005 |
| Intravenous fluid | 8 (4.3%) | 10 (18%) | 0.001 |
|  |  |  |  |

^a^ HIV screening was systematically performed in the public clinics only; ^b^ Missing blood pressure measurement in 36 patients; blood pressure was assumed to be within the normal range if missing; ^c^ Blood count values missing in 8 patients; ^d^ patients who died are included among patients with severe dengue.

Abbreviation: NA = not applicable.
